# Subsystem Hazard Analysis on an Offshore Waste Disposal Facility

**DOI:** 10.3390/ijerph17217755

**Published:** 2020-10-23

**Authors:** Sang-Ho Oh, Seung-Woo Kim

**Affiliations:** 1 Coastal Development and Ocean Energy Research Center, Korea Institute of Ocean Science and Technology, 385 Haeyang-ro, Yeongdo-gu, Busan 49111, Korea; 2 Department of Risk Assessment, Risk Solutions Inc., Seoul 04701, Korea; seungwookim76@gmail.com

**Keywords:** offshore waste disposal facility, hazard analysis, risk matrix, subsystem, environmental impact

## Abstract

Offshore waste disposal facilities are unique marine infrastructures that exist only in a few countries. Although the existing facilities in Japan and Singapore have been successfully operated in general, there have been no investigations on the probable hazards they pose on the environment. Considering this, conceivable hazards were identified for an offshore waste disposal facility that has recently been proposed in Korea. The causes and consequences of each of the identified hazards were analyzed to seek countermeasures for reducing the environmental impact in advance. Hazards of waste disposal facilities can be classified according to their design, construction, maintenance, operation, and site utilization. For these areas, except for site utilization, subsystem hazard analysis was performed. In the initial assessment, seven elements were found to be in the extreme risk zone, 30 were in the high-risk zone, and six were in the moderate-risk zone. After applying the alternative mitigation methods, the final risk assessment resulted in 27 moderate-risk and 16 low-risk elements. Therefore, it was confirmed that the potential risks of the proposed offshore waste disposal facility were within acceptable ranges.

## 1. Introduction

Offshore waste disposal refers to the final landfilling of stabilized inorganic solid waste, such as land and marine waste incineration materials. Although the demand for new waste landfill sites continues, waste disposal space is insufficient because of the imminent end of life in current landfill sites, and the difficulty in securing new landfill sites. Thus, an offshore waste disposal facility as a final waste disposal space was proposed as an alternative landfill site [1]. In Japan and Singapore, offshore waste disposal facilities are being operated to solve the lack of space for land waste disposal, while also creating eco-friendly marine spaces [2,3,4,5].

Since the 1990s, Japan has been conducting a thorough reclamation plan with local environmental surveys in advance, based on experience in the construction of multiple offshore waste disposal facilities [6,7]. Furthermore, based on the survey results, efforts have been made to minimize the impact on the marine environment by predicting environmental impacts and establishing measures to mitigate environmental pollution. In general, offshore waste disposal facilities in Japan have been successfully operated in the long term, through continuous monitoring, without major environmental problems or damage [8].

In Singapore, meanwhile, the volume of urban waste started to increase when the economy grew rapidly from the 1970s [9]. Hence, Singapore promoted the construction of its Semakau Landfill offshore facility by enclosing the sea between two islands. Since then, efforts have been made to restore the environment through monitoring and management plans to minimize potential impacts. Examples include restoring the mangrove forests and coral reefs that were destroyed during the construction of the Semakau offshore waste disposal facility [10,11].

In Korea, the necessity of constructing an offshore waste disposal facility as an alternative to land-based landfills has steadily increased, and accordingly, studies on the development of the core technologies and necessary institutional standards have been conducted [12,13,14]. However, due to various environmental and social conflict factors, such as pollution and the opposition of local residents to the construction of waste treatment facilities nearby, to date there is no decision or plan for an offshore waste disposal facility construction [8]. Because the facility is environmentally and socio-economically sensitive and there have been no previous construction cases in Korea, risk analysis is required to understand the expected risks associated with the construction of this facility. By examining the factors that will cause risks in advance, it is helpful to increase the social acceptance of this facility and to eventually build safer structures.

Risk analysis is often carried out regarding structures that have not been built in the past or are considered to be highly hazardous [15]. Although such an analysis has not been generalized for coastal or offshore structures, hazard analysis from an accredited international organization is mandatory when constructing an offshore plant [16,17,18,19,20]. Usually, such a risk analysis is carried out in accordance with the international standard code ISO 31000:2009 procedure [21]. Moreover, various hazard assessment studies are being conducted in relation to natural disasters and various human activities occurring on the coast and in the sea [22,23,24,25]. Nevertheless, to the best of our knowledge, studies on hazard evaluation have not been conducted thus far for offshore waste disposal facilities.

However, a study was recently completed to identify hazards that could occur during the construction and operation of an offshore waste disposal facility, analyzing the causes and effects of identified hazards specific to Korea [26]. A preliminary hazard list analysis (PHL) and preliminary hazard analysis (PHA) on offshore waste disposal facilities were performed, and hazard risks were evaluated. However, the previous study [26] was an analysis at the conceptual design level of an offshore waste disposal facility, which did not reflect the regional characteristics of the facility. Accordingly, the study was limited in that it did not address the specific design of an offshore waste disposal facility.

To overcome this limitation, in the present study, the subsystem hazard analysis (SSHA) was performed using specific design data for an offshore waste disposal facility. SSHA is a procedure performed when detailed design is available as it provides a more in-depth analysis on the hazards previously identified by PHA [15]. Since SSHA is carried out on more detailed design information, hazard elements of PHA are inherited or eliminated, and new hazard elements are also discovered. Therefore, SSHA refines identifying hazards, their associated causal factors, level of risk, and mitigating design measures.

The location of the offshore waste disposal facility on which the SSHA in this study was performed is inside the extension section of the dredged soil disposal area close to Incheon Songdo International City. In comparison with the previous PHA, hazard items are more specifically identified, and the causes and effects of the identified hazard items become clearer. In particular, since a populated city under development is located near the offshore waste disposal facility, considerations for minimizing the environmental impact were taken seriously. However, detailed hazard elements to the residence around the facility were not directly dealt with because this study conducted a hazard analysis mainly focusing on the structural risks of the offshore waste disposal facility.

## 2. Outline of Subsystem Hazard Analysis

**Figure 1:**
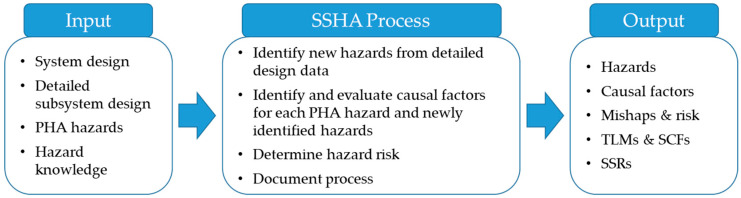
Overview of the subsystem hazard analysis (adapted and redrawn from [27]).

**Figure 2:**
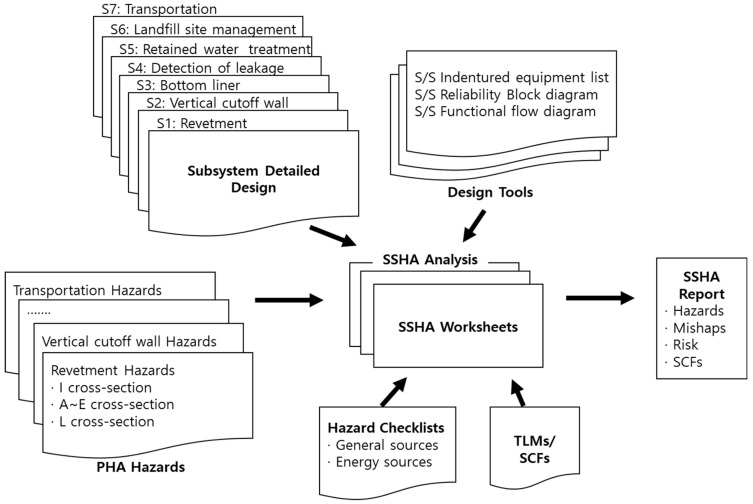
Conceptual diagram of the subsystem hazard analysis for the offshore waste disposal facility. PHA denotes preliminary hazard analysis, SSHA—subsystem hazard analysis, TLM—top-level mishaps, and SCFs—safety critical functions, respectively.

System safety identifies the potential hazards of the system and derives a measure to reduce each one. Hazard analysis (HA) focuses mostly on identifying hazards at the beginning of the entire process of system safety [15]. The identification of these hazards is carried out through the HA, and the SSHA is performed when basic and detailed design information is available. The purpose of hazard analysis at this stage is to accurately analyze the causes of the hazards identified in the PHA and to present specific risk mitigation measures. In addition, as the design progresses in detail, new hazards that were not recognized in the previous stage may be identified, and those previously identified may be removed. In other words, SSHA is a step that specifically expands PHA. Thus, the methodology is similar to PHA, however, more thorough results are obtained. Figure 1 and Figure 2 are an overview and the conceptual diagram of SSHA, respectively. The causes and effects of hazards are accurately assessed by comparing them to the hazard checklist based on PHA results and the detailed subsystem design. The core content here confirms how hazards of the previously performed PHA changed during the detailed design process, specifying the causal factors of hazards and their mitigation measures. In this process, the system’s top-level mishaps (TLMs), safety critical functions (SCFs), and system safety requirements (SSRs) are produced.

The detailed process of SSHA includes 10 steps. The first step is system definition, the second step is analysis planning, the third step involves the establishment of safety standards, the fourth step involves detailed designing and data collection (such as a hazard checklist), the fifth step requires the execution of SSHA, the sixth step is hazard evaluation, the seventh step is the proposal of a hazard reduction plan, the eighth step involves monitoring whether the presented hazard mitigation measures are effective as safety recommendations or system safety requirements, the ninth step is hazard tracking, and the tenth and final step is documentation.

The most important step in the above process is the fifth step of the SSHA. First, sub-divided subsystem elements are listed, elements of each list are evaluated, and the causal factors of any hazards are identified from the subsystem elements. At this time, the hazard risks identified in PHA are also analyzed in parallel. Furthermore, new hazards not identified in the PHA are identified through TLMs, SCFs, hazard checklists, and similar incident cases. When identifying all the hazards, the functional relationship, timing, and parallel functions of the subsystem elements should be understood.

## 3. The Offshore Waste Disposal Facility

### 3.1. The Facility of Investigation

An offshore waste disposal facility is a gravity-based structure that forms an outer revetment and stores waste inside it. The revetment protects the facility from maritime external forces such as waves and tsunamis. It should be a watertight structure to prevent any leaking of leachate. For this purpose, it is typically constructed on a seabed of clay deposit that effectively restricts any vertical flows through the seabed. Meanwhile, vertical cut-off barriers are formed by using the sheet piles that are installed along the inner side of the revetment to prevent any horizontal flows across the barrier. The space between the outer revetment structure and the sheet piles is typically filled with a backfill of rubble stones.

**Figure 3:**
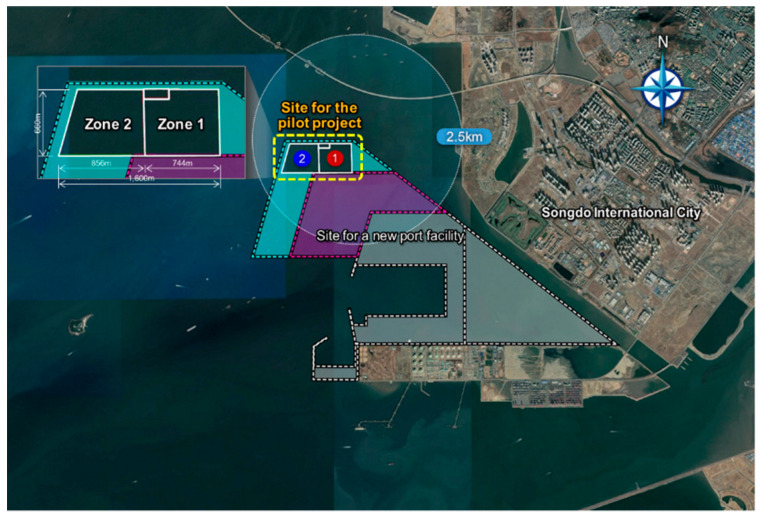
Location of the proposed offshore waste disposal facility.

In this study, SSHA was carried out for the proposed project of constructing an offshore waste disposal facility within the dredged soil disposal pond that is being built in the course of constructing the new Incheon port [12]. Figure 3 shows the location of the pilot project, which is very close to the new Incheon port in the south and Songdo International City in the southeast. The planned waste disposal facility is 1.6 km long and 0.6 km wide, and it is divided into two zones of almost equal areas. Once the facility is built, Zone 1 will initially be used for waste disposal, following which Zone 2 would be used for the same purpose when Zone 1 is almost filled with disposed waste.

By constructing a waste disposal facility here, construction costs can be reduced because a portion of the revetment of the disposal pond can be used as the outer revetment of the planned waste disposal facility. In addition, the sea transportation of waste may be unnecessary once this site is connected to the land in the future. On the other hand, there would be opposition from the residents because it is adjacent to Songdo International City, which is only 2.5 km from the residential town.

### 3.2. Subsystem of the Offshore Waste Disposal Facility

The SSHA of offshore waste disposal facilities must consider all the aspects of facility design, construction, maintenance, and operation. More specifically, hazards such as failures of the revetments, leakage of surface or vertical cut-off barriers, and the risks accompanied by waste transport should be carefully considered. Considering this, a hazard analysis was conducted by classifying the subsystem of the offshore waste disposal facility into the following seven major categories: revetment, vertical cut-off barrier, surface-cut-off barrier, leakage monitoring and detection, retained water treatment facility, landfill management, and waste transportation.

#### 3.2.1. Revetment

**Figure 4:**
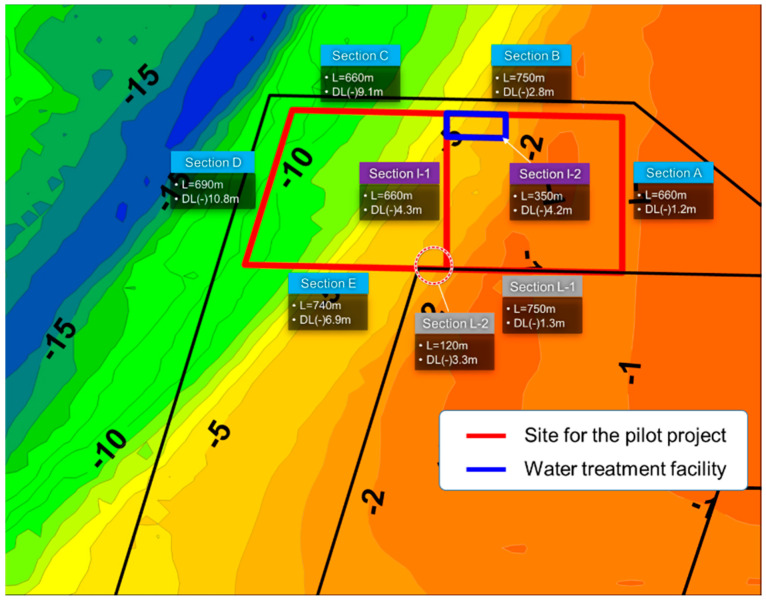
Details of the revetments constituting the proposed offshore waste disposal facility.

**Figure 5:**
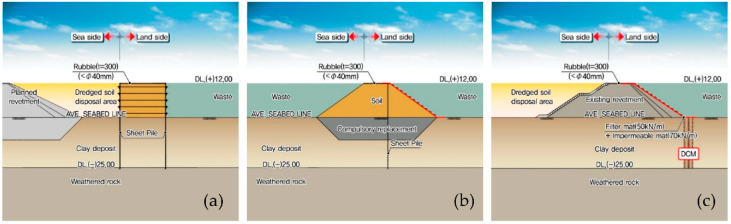
Cross-sections of the revetments constituting the offshore waste disposal facility: (**a**) the barrier revetment corresponding to Sections A–E; (**b**) the separating revetment corresponding to Section I; and (**c**) the connection revetment corresponding to Section L.

Figure 4 shows the plan view of the outer and inner revetments (marked using red lines) that constitute the proposed offshore waste disposal facility. Meanwhile, the black lines in Figure 4 indicate the revetment of the dredged soil disposal pond that is being built during the construction of the new Incheon port. Sections A to E are termed as barrier revetments because they act as a barrier that prevents the outflow of waste and retained water, rather than directly resisting the external forces from the ocean. The cross-section of these sections is composed of double vertical layers of sheet piles, as shown in Figure 5a.

Meanwhile, Section I is termed as a separating revetment because its function is to divide the inner space of the waste disposal facility into parts depending on the usage of the facility. Section I-1 divides Zones 1 and 2, as illustrated in Figure 3, whereas Section I-2 separates the space for the water treatment facility from Zone 1. As shown in Figure 5b, the cross-section of the separating revetment is a newly constructed rubble mound structure that includes vertical sheet piles in the middle and water-proof sheets on the slope.

Finally, Section L, termed as a connection revetment, utilizes the revetment of the dredged soil disposal pond and supplements it with only an additional function as a barrier. Accordingly, the soil strength is improved by means of deep cement mixing (DCM) and waterproof sheets are installed on the slope of the existing revetment.

#### 3.2.2. Vertical Cut-Off Barrier

For the barrier revetment (Sections A–E), the vertical cut-off barrier, which is composed of double-layer sheet piles, is applied as a means of preventing leachate movement. For the junction between the neighboring sheet piles, an expansive-type water stop material was used.

#### 3.2.3. Surface and Floor Cut-Off Barrier

A surface cut-off barrier was applied to the separating revetment (Section I) and the connection revetment (Section L), marked in the red thick lines on the slope of the revetment in Figure 5b,c. The surface cut-off barrier is composed of a filter mat and a double-layer waterproof sheet. In general, the permeability coefficient of the waterproof sheet ranges from 1.0 × 10^−2^ to 1.0 × 10^−4^. For the connection revetment, the DCM method was applied to the clay deposit at the toe of the revetment as illustrated in Figure 5c. Then, the waterproofing performance is very important at the interface of the seabed where the revetment toe and the DCM meet.

On the other hand, the floor cut-off barrier is typically not considered if the sea bottom has a low permeable layer, which satisfies the conditions of the permeability coefficient and thickness standards required for the landfill facility. If there exists an insufficient low-permeable layer underneath the seabed, a separate floor cut-off barrier treatment is requested. For this purpose, an expandable particle-type barrier material can be applied to form the floor barrier.

#### 3.2.4. Leakage Monitoring and Detection

Monitoring of water leakage in hydraulic structures or landfills is essential. In a waste landfill on land, the electric methods are commonly used for detecting damage to the barrier by installing electrodes on the waterproof sheet and measuring the electricity flow between two different electrodes [28]. If there is any leakage along the waterproof sheet, there should be significant electricity flow due to the insulation of the sheet being broken.

In the case of the offshore waste disposal facility in Tokyo Bay, Japan, the inspection of hazardous substances is carried out on a monthly basis at the wells around the revetment that are prepared for water quality testing. The leakage of retained water is also indirectly monitored by placing sand tubes between concrete blocks in the external revetment and monitoring whether there is any loss of sand from the sand tubes.

In this study, leakage monitoring and detection has been considered an independent subsystem of the offshore waste disposal facility. This deals with the methodology itself detecting a leakage when either the vertical barrier or surface and floor barrier are damaged. If damage occurs to a barrier and it is not detected, the hazard posed by the waste disposal facility may increase. Therefore, it is reasonable to be considered as an independent subsystem apart from the barrier structures.

#### 3.2.5. Retained Water Treatment Facility

The retained water treatment facility, represented using a blue box in Figure 4, was designed to be located in Zone 2. This facility treats the retained water in Zone 2 as well as Zone 1. The maximum capacity of the treatment facility is determined as 4000 m^3^/day, taking into account the daily amount of carry-in waste. When only Zone 1 is used, it will treat up to 2000 m^3^/day of the retained water. When Zone 2 is used in addition to Zone 1, the capacity will be extended to up to 4000 m^3^/day.

#### 3.2.6. Landfill Management

Landfill management refers to how the waste disposal facility is managed while it is being landfilled with carry-in waste. As shown in Table 1, there exist various monitoring items that are required for efficient landfill management. In order to effectively monitor the items listed in Table 1, fixed or mobile sensing methods can be applied depending on the measurement purpose and preferences. A fixed sensing method is used when measuring the deformation of the revetment or change of water level inside and outside the disposal site. Meanwhile, a mobile sensing method is adequate for measuring parameters such as the landfill height or water quality, which are required to be measured in different locations over a wide area of the facility.

#### 3.2.7. Waste Transportation

The proposed site of the offshore waste disposal facility is located where access by land is possible. Accordingly, direct waste disposal to the waste disposal facility through land transportation can be carried out, which does not require a carry-in base for sea transportation. In this case, the hazards associated with water transportation can be eliminated. In addition, civil complaints due to scattering dust and odor, which typically take place in the vicinity of the carry-in base, can be significantly reduced.

## 4. Procedures of Subsystem Hazard Analysis

### 4.1. Selection of Relevant Hazard Checklists

A hazard checklist commonly includes elements such as energy sources, hazardous functions, hazardous operations, hazardous materials, undesired mishaps, and lessons learned from similar type systems [15]. A hazard checklist by itself is not complete and does not include all hazardous items. It helps in identifying potential hazards through past examples. Therefore, different hazard checklists can be established depending on the analysis. The bulleted list below shows the hazard checklists considered in this study, which are relevant to the hazard analysis of offshore waste disposal facilities:

- Structural damage or failure;
- Leakage;
- Contamination;
- Corrosion;
- Toxicity;
- Weather and environment.

### 4.2. Identification of TLMs

TLMs refer to an important hazardous accident caused by one or more causes. It is a concept used to collect various potential hazards that lead to the same TLM outcome. TLM defined in the previous PHA study [25] mostly focused on safety concerns on external structures and cut-off barriers. Among them, some TLMs such as the sliding and overturning of caisson are removed in the present SSHA study because caisson is no longer adopted as an external structure of the subsystem of the proposed offshore waste disposal facility. On the other hand, additional hazards such as suspended particles or traffic congestion need to be included considering that the proposed site for the pilot project is close to a populated city under development. Meanwhile, the hazards related to energy sources include mechanical and electrical equipment of the retained water treatment facility. However, the hazards from this equipment were not included in TLMs because they are of relatively low importance in the design. The bulleted list below illustrates the TLMs considered in this study:

- Corrosion of sheet piles;
- Deformation and damage at the joint of sheet piles;
- Insufficient embedded depth of sheet piles;
- Damage to the waterproof sheets of cut-off barriers;
- Difficulty in operating a land base into which waste is carried;
- Suspended particles in the air;
- Leakage of leachate to the sea.

### 4.3. Assessment of IMRI and FMRI

Table 2 shows a worksheet that is commonly used in SSHA [15]. In the table, ① is the number of subsystem hazards that are identified. Entry ② represents a specific hazardous element that is expected to occur, and ③ represents the initiating mechanism by which hazardous elements lead to mishaps. Entry ④ indicates the consequence when a hazard has occurred. Entry ⑤ is an initial mishap risk index (IMRI), which serves as a qualitative indicator of the importance of the identified hazards. Entry ⑥ indicates a possible preventive measure to eliminate or mitigate the identified hazard. Lastly, ⑦ is a final mishap risk index (FMRI), a qualitative indicator of the action taken to reduce the identified hazard.

IMRI and FMRI use the US Department of Defense’s system safety practice standard, MIL-STD-882D, which has widely been used internationally [27]. In MIL-STD-882D, the hazards are classified into four categories according to the degree of severity and five categories according to the probability of occurrence, as shown in Table 3. IMRI and FMRI are represented as a combination of a single index from each column in the table. For instance, a critical hazard that occasionally occurs is denoted as 2C.

When IMRI and FMRI are assessed in this study, the hazard severity was considered based on the importance and extent of the damage. Catastrophic damage corresponds to damage that leads to the long-term shutdown of the offshore waste disposal facility, such as the leakage of leachate to the marine environment due to the extensive damage of the external revetment. Critical damage refers to a case in which the operation of the offshore waste disposal facility is suspended for considerable time due to the severe degradation of the cut-off barrier performance caused by the deformation of the outer revetment, the breakage of vertical and surface barriers, or the failure of the retained water treatment facility. Marginal damage means a temporary suspension of the facility operation influenced by local damage to the cut-off barrier, problems with landfill management, or a shortage of capacity to process the retained water.

Meanwhile, hazard mitigation or reduction measures, corresponding to entry ⑥ in Table 2, are generally presented as forms of system improvement requirements. Hazard mitigation measures can be classified into four stages in terms of priority. First, it is ideal to remove hazards from the stage of system design. If it is difficult to change the system design, however, supplementary safety devices should be applied alternatively. Third, if the application of safety devices is unnecessary or their effects on improving system safety are limited, the introduction of an alarm system can be the next alternative. Finally, non-facility measures, such as education and training on safety, can be implemented in addition to the measures focusing on mitigating the hazards of facilities.

## 5. Results of Subsystem Hazard Analysis

### 5.1. Mishap Risk Indices and Methods of Mitigation

As a result of the hazard analysis on the seven subsystems of the offshore waste disposal facility, 43 potential hazardous elements were identified, as listed in Table 4. The severity and probability of each hazardous element were evaluated through a workshop in which the experts from design firms, research institutes, and relevant authorities have participated. Then, it was possible to determine the risk level of each hazardous element by following the risk assessment method described in MIL-STD-882E [29]. More specifically, the appropriate severity category for a given hazard element was determined by assessing the potential for death, injury, environmental impact, or monetary loss. In addition, the appropriate probability level was determined by evaluating the likelihood of occurrence of a mishap. It is desirable to use relevant quantitative data as a basis for objectively assessing the severity and probability. When quantitative data are not available, a single opinion is obtained through the Delphi analysis or a second-round workshop after the initial evaluation by each expert.

**Figure 6:**
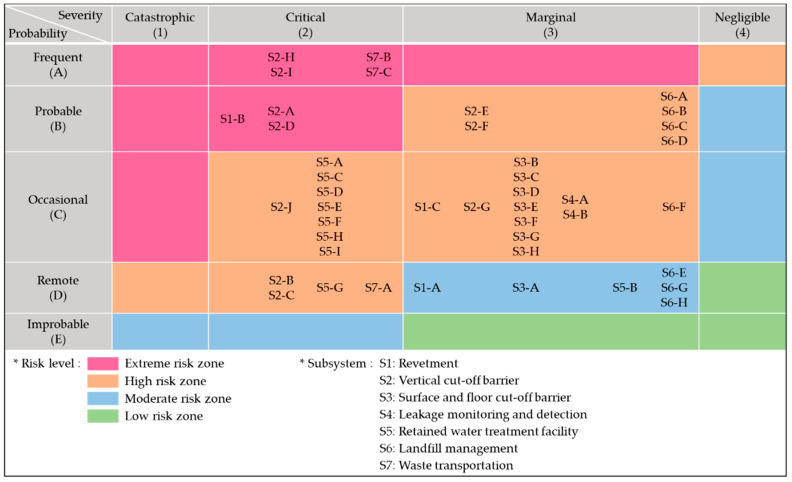
Risk assessment matrix of initial subsystem hazard analysis (SSHA) of the offshore waste disposal facility.

The results obtained from the above procedure are summarized in Figure 6 as a form of risk assessment matrix. As shown in Figure 6, seven out of 43 elements were classified into the extreme risk zone. Among the seven extremely risky hazardous elements, one is related to the subsystem of the revetment (S1), four to the vertical barriers (S2), and two to waste transportation (S7). Meanwhile, 30 elements were classified into the high-risk zone, and the remaining six were in the moderate risk zone.

For every identified and classified hazard, necessary measures should be sought to eliminate or reduce the risk in advance. Table 5 summarizes a general principle for seeking a measure required for reducing or controlling the potential hazards depending on the associated risk level. The ultimate goal is always to find the most appropriate solution to eliminating the hazard. When it is impossible to completely eliminate a hazard, the assessed risk should be reduced to the lowest acceptable level with the approval or agreement of the authorities or parties, after taking account all of the relevant constraints such as cost, time, and other factors [29]. In other words, when removing a hazard is difficult, hazard management is required.

Table 6 shows the example worksheet presenting IMRIs, mitigation measures, and FMRIs of the seven hazardous elements that were classified to be extremely risky. For the remaining hazardous elements corresponding to high or moderate risk, similar worksheets were created, however, they were omitted as they were considered excessively lengthy to be included in this paper. Basically, the process of evaluating FMRI is similar to that of IMRI. For every hazard element evaluated as extreme or high risk in IMRI, a mitigation measure was suggested for reducing the associated risk to an acceptable range. Then, the severity and probability after applying the mitigation measure were evaluated by experts who conducted assessment of IMRIs by following the same procedure described in Section 5.1. If the risk of a hazard element was not reduced to the lowest acceptable level by the firstly proposed mitigation measure, this process was repeated until it was satisfied.

**Figure 7:**
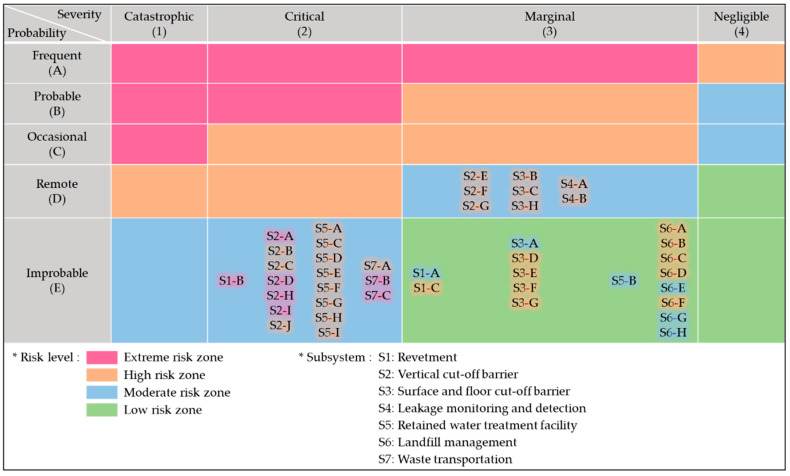
Risk assessment matrix of the final subsystem hazard analysis (SSHA) of the offshore waste disposal facility. Blurred background colors indicate the results of the initial SSHA.

The comparison of IMRIs and FMRIs in Table 6 shows that the risk levels of the hazardous elements have been apparently reduced by applying the mitigation measures. This fact is more easily confirmed in Figure 7, which shows the modified risk assessment matrix of the offshore waste disposal facility after applying the mitigation measures. The items identified in the extreme risk zone and the high-risk zone in Figure 6 have been moved to the moderated risk zone and the low-risk zone in Figure 7, respectively. Hence, all the hazards associated with extreme or high risks in IMRIs have been evaluated as moderate or low risks in FMRIs. This indicates that the risks of the originally proposed design have been significantly mitigated through SSHA. By applying the mitigation measures to the original design, the overall stability of the design was improved, especially with regard to the safety of the revetment and the cut-off barriers, which are the core structures constituting the offshore waste disposal facility. Note that the FMRIs of all the identified hazardous elements were evaluated through a process similar to IMRIs, by holding a workshop with various members from relevant organizations.

### 5.2. Design Modification by Reflecting on SSHA

**Figure 8:**
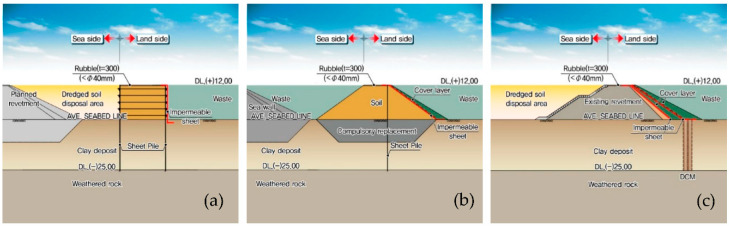
Cross-sections of the revetments constituting the offshore waste disposal facility that have been modified based on the subsystem hazard analysis. (**a**) The barrier revetment corresponding to Sections A–E; (**b**) the separating revetment corresponding to Section I; and (**c**) the connection revetment corresponding to Section L.

As described in Section 5.1, the risks of the offshore waste disposal facility were analyzed and measures to mitigate such risks were suggested through SSHA. Reflecting on this, the original design was subjected to a complete revision, from which some parts of the design were changed. Figure 8a‒c show the modified cross-sections of the barrier revetment, separating revetment, and the connection revetment from the initial design shown in Figure 5a‒c.

The major risks of barrier revetment (Sections A‒E) were the poor construction of the barrier for some reasons and the deterioration of the cut-off performance that would lead to a mixture of waste and dredged soil. To mitigate these types of risks, additional waterproof sheets were placed along the interior sheet piles in the updated design as shown in Figure 8a.

Meanwhile, the critical risks of the separating revetment (Section I) were the failure or collapse of the revetment due to the landfill overload or other unexpected external forces and exceedance of the capability of the waste treatment facility. Such risks have been reduced by placing a supplementary cover layer on the inner slope of the separating revetment and waterproof sheets underneath the cover layer. Figure 8b shows the modified design according to these changes.

Finally, the primary risk of the connection revetment (Section L) was the lack of continuity at the connection between the improved soil by DCM and the cut-off barrier on the revetment slope. If there exists any discontinuity, leachate shall be leaked to the dredged soil disposal pond through the revetment. To prevent this risk, a relatively thick cover layer was placed with waterproof sheets in the middle, as shown in the updated design in Figure 8c.

As illustrated in Figure 8, improved design outputs were obtained through SSHA by updating the original design where hazard elements not recognized by designers in the early stages of the design were included. In this context, the results presented in this study are meaningful in that they introduce a method to objectively recognize and manage the risks associated with the construction of the offshore waste disposal facility. In addition, the design drawings derived through this study were made at the conceptual design level but intrinsically are comparable to the basic design level. Therefore, they will be practically used as a key material for future detailed design when the construction of the facility is determined and a full-fledged demonstration project becomes visible.

## 6. Conclusions

This study provided the result of SSHA for an offshore waste disposal facility, which, to the best of our knowledge, has not previously been presented in any literature so far. The hazard analysis was performed for a detailed design of the proposed offshore waste disposal facility that was planned to be constructed near the new Incheon port, Korea. According to the IMRIs that were assessed by SSHA, seven hazard elements corresponded to extreme risks, 30 high risks, and seven moderate risks. After applying the risk mitigation measures, however, there were no hazard elements associated with extreme or high risks. All the hazard elements came into moderate or low risks, which can be controlled or managed adequately.

Therefore, this study shows that potential hazardous elements of the offshore waste disposal facility can be identified in advance through SSHA and countermeasures can be established to eliminate or mitigate the identified risks. However, it remains uncertain as to whether the proposed offshore waste disposal facility near Songdo International City can actually be built. Because such a facility has never been built in Korea, even if it can be constructed with only marginal or negligible influence on the environment, it may take a long time to obtain the consent and agreement of the local residents. In this context, when the actual construction plan is dealt with more specifically, it is necessary to perform an additional hazard analysis that focuses more on the risk of the residence.

## Figures and Tables

**Table 1:** Monitoring items and remarks for landfill management.

Monitoring Items	Remarks
Changes in water level inside and outside the offshore disposal facility	Performance of the cut-off barrier can be checked by monitoring changes in water level inside and outside the facility. It is necessary to confirm that the water level inside the facility is not influenced by the adjacent tide level.
Revetment deformation due to ground settlement	It is necessary to measure the displacement of revetment on the ground as well as underwater. Monitoring ground settlement is also required by installing inclinometers and settlement gauges.
Landfill height change	Landfill height should be distributed as evenly as possible within the disposal facility for stable landfill management.
Water quality inside and outside the facility	Measurement of dissolved oxygen (DO), pH, and other water quality indices is requested at least once every 6 months over at least two locations.

**Table 2:** Worksheet used for the subsystem hazard analysis. IMRI—initial mishap risk index, FMRI—final mishap risk index.

No.	Hazard	Causes	Consequences	IMRI	Mitigation	FMRI
①	②	③	④	⑤	⑥	⑦

**Table 3:** Mishap risk indices according to MIL-STD-882D [27].

Severity	Probability
(1) Catastrophic	(A) Frequent
(2) Critical	(B) Probable
(3) Marginal	(C) Occasional
(4) Negligible	(D) Remote
	(E) Improbable

**Table 4:** The list of severities and probabilities of all 43 identified hazards.

Code	Hazard	Severity	Probability
S1-A	Cracking and collapse of the separate revetment	3	D
S1-B	Circular slip failure of the revetment	2	B
S1-C	Displacement of the revetment due to waste disposal	3	C
S2-A	Corrosion of barriers made of steel	2	B
S2-B	Deformation and damage of joints caused by an earthquake	2	D
S2-C	Deformation and damage of joints during landfill	2	D
S2-D	Deformation and damage of joints due to landfill pressure	2	B
S2-E	Insufficient stability of mortar at joints caused by poor filling	3	B
S2-F	Degradation of stability of mortar at joints caused by aging	3	B
S2-G	Poor construction of joints	3	C
S2-H	Insufficient depth of barriers due to poor construction	2	A
S2-I	Insufficient depth of barriers due to heterogeneity of ground	2	A
S2-J	Degradation of cut-off performance at joints	2	C
S3-A	Locally permeable ground	3	D
S3-B	Damage of waterproof sheets due to the poor fusion of adhesive parts	3	C
S3-C	Damage of waterproof sheets due to landfill weight	3	C
S3-D	Damage of waterproof sheets due to slip on the slope	3	C
S3-E	Degradation of cut-off performance of the separate revetment	3	C
S3-F	Degradation of cut-off performance of the connection revetment	3	C
S3-G	Floating of waterproof sheets due to lack of sufficient weight	3	C
S3-H	Degradation of cut-off performance of the barrier revetment	3	C
S4-A	Failure of detecting leakages of leachate	3	C
S4-B	Failure of confirming the location where leakages occur	3	C
S5-A	Failure of treating retained water	2	C
S5-B	Degradation of the capability of retained water treatment	3	D
S5-C	The outflow of retained water to the sea	2	C
S5-D	Exceedance of retained water greater than treatment capacity	2	C
S5-E	Fire	2	C
S5-F	Flooding	2	C
S5-G	Electric shock from high voltage	2	D
S5-H	Wave overtopping	2	C
S5-I	Deterioration of employee’s health	2	C
S6-A	Malfunction of the sensors measuring water level	3	B
S6-B	Malfunction of the sensors measuring water quality	3	B
S6-C	Exceedance of measurement range of the sensors	3	B
S6-D	Power supply interruption to the sensors	3	B
S6-E	Malfunction of the movable sensing device	3	D
S6-F	Errors in the communication signal	3	C
S6-G	Inability of collecting sensing data	3	D
S6-H	Failure of operation and management system	3	D
S7-A	Collison of a ship to the revetment	2	D
S7-B	Suspended particles in the air	2	A
S7-C	Traffic congestion	2	A

**Table 5:** Measures required for reducing or controlling the hazards according to the associated risk level [29].

Risk Level	Measure
Extreme	Shall be eliminated
High	Shall only be accepted when risk reduction is impracticable and with the agreement of the authority
Moderate	Acceptable with adequate control and the agreement of the authority
Low	Acceptable with/without the agreement of the authority

**Table 6:** Extremely hazardous elements identified by subsystem hazard analysis (SSHA) and measures to mitigate them. IMRI—initial mishap risk index, FMRI—final mishap risk index.

No.	Hazard	Cause	Consequence	IMRI	Mitigation	FMRI
S1-B	Circular slip failure of the revetment	Excessive surface load on the separating revetment	Collapse of the revetment	2B	Modify the design of the separating revetment to satisfy the safety standards by reinforcing the fore slope of the revetment	2E
S2-A	Corrosion of barriers made of steel	Exposure to seawater	Cracks in the barrier and deterioration of cut-off performance	2B	Apply cathodic protection method or use special steels for anti-corrosion	2E
S2-D	Deformation and damage of joints due to landfill pressure	Pressures from the landfilled waste	Cracks in the barrier and deterioration of cut-off performance	2B	Apply a double layer waterproof sheets on the joints or use sheet piles implementing fail-safe technology	2E
S2-H	Insufficient depth of barriers due to poor construction	Uncertainty associated with the construction of vertical barriers	Failure of barriers or deterioration of cut-off performance	2A	Monitor the driving depth of vertical barriers and check verticality during construction	2E
S2-I	Insufficient depth of barriers due to heterogeneity of ground	Poor construction of vertical barriers influenced by the heterogeneity of ground	Failure of barriers or deterioration of cut-off performance	2A	Apply longer barriers than required to have extra driving depth considering the associated uncertainty	2E
S7-B	Suspended particles in the air	Waste transportation and unloading to the disposal facility	Problems with the health and well-being of local residents	2A	Apply a shield to the transportation vehicles and build a special device to minimize particle suspension during transportation and unloading	2E
S7-C	Traffic congestion	Waste transportation on land	Inconvenience and uneasiness to local residents	2A	Make a detour away from the residential area and transport waste avoiding crowded hours	2E
